# Moxibustion for stable chronic obstructive pulmonary disease: A protocol for systematic review and meta-analysis

**DOI:** 10.1097/MD.0000000000025713

**Published:** 2021-04-30

**Authors:** Yanping Wang, Mingru Huang, Liping Tang, Lingxia Xu, Jiangfeng Wu, Fei Wang, Ying Zhang

**Affiliations:** a Postgraduate Education, Jiangxi University of Traditional Chinese Medicine; b Respiratory Department, Jingdezhen Hospital of Traditional Chinese Medicine; c Academician Workstation; d Institute of Traditional Chinese Medicine, Jiangxi University of Traditional Chinese Medicine, Jiangxi, China.

**Keywords:** chronic obstructive pulmonary disease, moxibustion, protocol, systematic review

## Abstract

**Background:**

There is no optimal treatment to alleviate the decline of lung function in the stable phase of chronic obstructive pulmonary disease (COPD). The effectiveness of moxibustion as an adjunctive treatment for COPD in the stable phase has been reported clinically, but the conclusions on efficacy and safety have not been unified. This study will systematically evaluate the efficacy and safety of moxibustion on the treatment of COPD in the stable phase, providing clinical-based evidence

**Methods:**

We will systematically search 7 literature databases and 2 clinical trial registration platforms. The searching time will be conducted from the establishment of databases to March 31, 2021, regardless of language. We will include the randomized controlled trial (RCT) evaluation of moxibustion combined with basic therapy vs basic therapy alone for the treatment of stable COPD. We will assess the risk of bias for individual RCTs using the Cochrane Handbook 5.1.0 evaluation tool. The primary outcome is forced expiratory volume in 1 second/forced vital capacity. The secondary outcomes include forced expiratory volume in 1 second, forced vital capacity, six-minute walking distance, COPD assessment test score, maximum ventilation, response to treatment, and incidence of adverse events. We will collect the effective data of individual RCT through systematic analysis of the random effect model. Heterogeneity will be tested by Cochran Q test and *I*-squared statistics. Two subgroup analyses will be performed to explore the sources of heterogeneity based on clinical experience. Excluding RCTs with a high risk of bias, fixed-effect model will be used for sensitivity analysis to test the robustness of the meta-analysis results. The publication bias will be assessed by funnel plot and Egger test.

**Results:**

This study will provide systematic evidence on the efficacy and safety of moxibustion on the treatment of patients with stable COPD through strict quality assessment and reasonable data synthesis. We hope that the results will be submitted to a peer-reviewed journal for publication.

**Conclusion:**

This systematic review will provide the best current evidence for the adjuvant treatment of stable COPD with moxibustion.

**INPLASY registration number:**

INPLASY202140047.

## 1 Introduction

Chronic obstructive pulmonary disease (COPD) is a lung disease characterized by persistent airflow limitation, with typical symptoms such as chronic and progressive dyspnea, cough, and sputum.^[1]^ COPD prevalence and mortality are higher, and the World Health Statistics 2017 shows that there are 877 million patients with COPD global wide and the prevalence rate is about 11.7%. There are approximately 3.5 million deaths caused by COPD each year,^[2]^ which is the third leading cause of death and the fifth leading cause of disease burden worldwide in 2020.^[3]^ Due to the increase in COPD risk factors caused by environmental pollution and the aging of the world population, WHO expects that by 2030 approximately more than 4.5 million people worldwide will die each year from COPD and its related diseases.^[4]^

The treatment principle of western medicine in stable COPD is to alleviate or prevent the decline of lung function using bronchodilators, glucocorticoids, mucolytics, antioxidants, and immunomodulators to improve the quality of patient survival and reduce the risk of acute exacerbation and mortality.^[5]^ Although drugs can temporarily alleviate the pain of the patients, drug dependence is strong. In severe cases, osteoporosis and gastrointestinal bleeding may occur. Therefore, researchers are still exploring new therapies that can help improve lung function. Moxibustion has been reported to be tried as an adjunctive treatment for stable COPD patients in a randomized controlled trial (RCT), which can relieve symptoms such as cough and chest tightness and improve the life qualities of COPD patients.

Traditional Chinese medicine elaborates on the characteristic of moxibustion: “the failure of medicine and the failure of needles must be moxibustion.” Moxibustion is mainly applied to stimulate the skin by burning with the heat generated by moxa directly or indirectly on specific acupuncture points or areas to unblock the meridians and regulate the body's function.^[6]^ Moxibustion is gentle, simple, long-lasting and safe without side effects, and has been widely used in the treatment of chronic lung diseases such as asthma and allergic rhinitis.^[7,8]^ Moxibustion has received much attention in the adjuvant treatment of stable COPD, and the results of a clinical study showed that mild moxibustion significantly improved clinical outcomes, enhanced lung function, improved dyspnea, and improved the quality of life in patients with stable COPD compared with the control group.^[9]^ Another result also showed that moxibustion could reduce serum IL-32 and caspase-1 levels in patients with stable COPD, and its mechanism of action might be related to the reduced the chronic inflammatory response of patients.^[10]^

Currently, there are available RCTs of moxibustion for the treatment of stable phase COPD, most of which are tested with small sample sizes. The efficacy of individual trial tests may be insufficient, and there is no systematic review on this topic. Therefore, from the perspective of evidence-based medicine, this study will comprehensively search and include all currently available RCTs of moxibustion for stable COPD, summarize the efficacy and safety of moxibustion for stable COPD, and provide high-level evidence for clinical application.

## 2 Methods

### 2.1 Study registration

This protocol was registered on the INPLASY platform. The registration number is INPLASY202140047 (URL= https://inplasy.com/inplasy-2021-4-0047/). We reported the protocol according to the Preferred Reporting Items for Systematic Review and Meta-Analysis Protocols (PRISMA-P) statement.^[11]^

### 2.2 Inclusion and exclusion criteria

#### 2.2.1 Types of studies

Trials will be considered eligible if they are RCTs, cohort trial studies or case-controlled studies. Animal studies, narrative reviews, conference abstracts, letters, literature providing duplicate data from the same trial, and failing to extract the required outcome will be considered ineligible.

#### 2.2.2 Types of participants

This study will include patients with a definite diagnosis of COPD in stable stage, whose symptoms of cough, sputum and shortness of breath are stable or mild and the disease has largely returned to its preacute exacerbation state.^[12]^ We will include patients ≥18 years old, with no restriction on patients gender or race. We will exclude acute COPD and COPD combined with other respiratory diseases, such as asthma, respiratory failure, and so on.

#### 2.2.3 Types of interventions and controls

The intervention measures of the experimental group will be moxibustion combined with conventional western medicine treatment or moxibustion alone. There will be no restrictions on the course, time, and point selection of moxibustion. Moxibustion combined with other traditional Chinese medicine treatments (e.g., acupoint application, cupping, massage, acupuncture, etc) will be excluded.

#### 2.2.4 Types of controls

The control group will be treated with conventional western medicine alone, such as bronchodilators, glucocorticoids, mucolytic agents, antioxidants, and immunomodulators.

#### 2.2.5 Types of outcomes

##### 2.2.5.1 Primary outcomes

The primary outcome is forced expiratory volume in 1 second/forced vital capacity.

##### 2.2.5.2 Secondary outcomes

The secondary outcomes are included:

1. Forced expiratory volume in 1 second (FEV_1_);
2. Forced vital capacity (FVC);
3. Maximum volume;
4. Six-minute walking distance (6-MWD)^[13]^;
5. COPD assessment test score^[14]^;
6. Response to treatment according to recognized classification criteria, such as Guiding Principles for Clinical Research of New Chinese Medicines;
7. Adverse events.

### 2.3 Search methods for the identification of studies

#### 2.3.1 Data sources

We will use electronic and manual search. The search time will be set from the establishment of databases to March 31, 2021, and there is no limit to the languages. The supplementary search of the reference list of included studies and relevant systematic reviews will identify as potentially eligible literature. We will search 7 databases (PubMed, EMBASE, Cochrane Library, Chinese Biomedical Literature Database, China National Knowledge Infrastructure, VIP Database, Wanfang Database). At the same time, we will search 2 clinical trial registration platforms: clinicaltrials.gov and Chinese Clinical Trial Registry.

#### 2.3.2 Search strategy

We will use a combination of medical subject headings and free words on the search strategy. Searching terms will include moxibustion, COPD, emphysema, respiratory insufficiency, lung, trachea, etc. Take PubMed as an example, the search strategy is described in detailed in Table 1.

**Table 1:** Search strategy in PubMed.

No.	Search terms	No.	Search terms
1	chronic obstructive pulmonary disease [mh]	14	#11 OR #12 OR #13
2	pulmonary disease[tw]	15	randomized controlled trial[mh]
3	chronic obstructive [tw]	16	controlled clinical trial [tw]
4	chronic obstructive airway disease [tw]	17	Randomized [tw]
5	airflow obstructions [tw]	18	Trial [tw]
6	chronic airflow obstruction [tw]	19	Contrast [tw]
7	emphysema [tw]	20	Groups [tw]
8	pulmonary emphysema [tw]	21	#15 OR #16 OR #17 OR #18 OR #19 OR #20
9	respiratory insufficienty[tw]	22	Animals [mh]
10	#1 OR #2 OR #3 OR #4 OR #5 OR #6 OR #7 OR #8 OR #9	23	Humans [mh]
11	Moxibustion [mh]	24	10 AND 14 AND 21
12	Moxa [mh]	25	22 NOT 23
13	Moxibustion [tw]	26	24 NOT 25

### 2.4 Data collection and analysis

#### 2.4.1 Study selection

**Figure 1:**
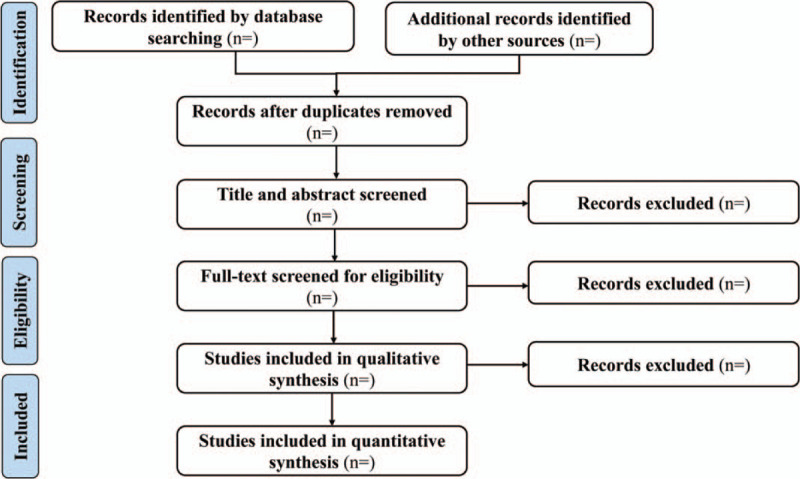
PRISMA-style flow chart of literature screening. CBM = Chinese Biomedical Literature Database, CNKI = China National Knowledge Infrastructure.

Two reviewers independently cross-screen the literature according to predetermined criteria, and will firstly eliminate irrelevant literature by reading the titles and abstracts. Then the remaining literature will read again to determine that they meet all the inclusion criteria. If there is any disagreement in the process of literature screening and extraction, it will be resolved by discussion of the 2 reviewer or invitation of a third reviewer to decide. The literature screening flow chart will be shown in a PRISMA-style flowchart (Fig. 1).

#### 2.4.2 Data extraction

Two reviewers will use predesigned Microsoft Excel to independently, standardly and repeatedly extract the data included in each study, and extract the following contents: first author, publication time, inclusion and exclusion criteria, sample sizes, intervention measures, age, gender, course of disease, course of treatment, FEV_1_/FVC, FEV_1_, FVC, maximum volume, 6-MWD, COPD assessment test score, response to treatment, and adverse events. We will compare the data extracted by 2 reviewers, and the differences will be resolved through consensus.

#### 2.4.3 Risk of bias assessment

Two reviewers will evaluate the risk of bias in the included studies and cross-checked the results. If they disagree, they will discuss and resolve with a third reviewers. The Cochrane bias risk assessment tool will be used to evaluate the included RCTs.^[15]^ Six items will be assessed, including random allocation method, allocation concealment, implementer blind method, result evaluator blind method, result data completeness, selective reporting, and other sources of bias. Each item ill be rated as “low bias,” “high bias,” or “uncertain.”

#### 2.4.4 Managing missing data

If the included studies lack relevant data, we will try to contact the first author or corresponding author of the article by email to obtain the required data. If the required information could not be obtained, the data will be excluded from the analysis and explained in the discussion section.

#### 2.4.5 Data synthesis

We will use STATA statistical software version 12.0 (StataCorp, College Station, TX) for statistical analysis. We will use the odds ratio for the combined effect size dichotomous data, and the weighted mean difference for continuous data. If the units are inconsistent and cannot be converted uniformly between the studies, the standardized mean difference will be used. For each effect size, its point estimate and 95% confidence intervals will be calculated.

#### 2.4.6 Assessment of heterogeneity

We will use the χ^2^ test to determine whether there is statistical heterogeneity between the included studies, and use *I*^2^ to quantitatively determine the heterogeneity. When *I*^2^ < 50% and χ^2^ test *P* ≥ .10, the heterogeneity is considered to be acceptable; *I*^2^ ≥ 50%, *P* < .10 is high heterogeneity, and the source of heterogeneity will be analyzed by subgroup analysis.

#### 2.4.7 Subgroup analysis

According to the subgroup analysis guide,^[16]^ we will explore the following subgroup hypotheses in advance to test the interaction between each subgroup *P* value:

1. type of moxibustion (direct moxibustion vs indirect moxibustion);
2. course of treatment (≥4 weeks vs <4 weeks).

#### 2.4.8 Sensitivity analysis

We will perform 2 types of sensitivity analysis:

1. Reanalyze the meta-analysis after excluding the relatively poor quality literature from the included studies, and compare whether there are significant differences between the combined effects before and after.
2. Using different effect model (fixed effect vs random effect) to calculate the effective combined value point estimation and interval estimation.

#### 2.4.9 Assessment of publication bias

We will use RevMan v5.3 funnel chart to comprehensively evaluate publication bias for the outcomes of the included studies ≥10. Combined with the Egger test correlation test, if *P* > .05, there is no publication bias and the funnel chart is symmetric; otherwise, there is publication bias and the funnel chart is asymmetric.

#### 2.4.10 Assessment of the quality of evidence

We will evaluate the quality of evidence for each outcome based on the Grading of Recommendations, Assessment, Development and Evaluation (GRADE) framework.^[17]^ The quality of the evidence will be rated according to the following 5 aspects, including risk of bias, indirectness, inconsistency, imprecision, and publication bias. A funnel chart check of standard errors and effective estimates will be performed for publication bias and small study effects.

### 2.5 Ethics and dissemination

This study will not involve personal or human test data, so ethical approval will not be required. Our aim is to publish research results in peer-reviewed journal.

## 3 Discussion

Various treatment of COPD all relieve symptoms to a certain extent and reduce the acute onset of COPD, but there is no effective way to eradicate COPD. Going throughout the clinical practice guidelines at home and abroad, the focus on the stable phase of COPD is how to effectively control the disease, slow the development of the disease and the deterioration of lung function, and improve the quality life of the patients. Moxibustion, as one of the adjuvant treatments in stable COPD, is widely used in stable COPD and show certain curative effects.

This study will comprehensively search multiple original databases and include relevant RCTs. By strict literature screening, data extraction and quality evaluation, detailed summary and analysis of 6-MWD, total effective rate COPD assessment test scores and lung function, and the included RCT, we will be conducted a rigorous risk assessment of bias. Although there is a systematic review of acupuncture and moxibustion treatment of COPD,^[18]^ all acupuncture and moxibustion RCTs are included, it did not stage COPD patients and did not conduct subgroup analysis of moxibustion, causing the heterogeneous results. This article focuses on the using of moxibustion to assist in the treatment of stable COPD and comprehensively evaluate its efficacy and safety.

The potential limitation of this study is that the possibly high risk of bias included in the study may affect the reliability of the results. Future large samples should be carried out with rigorous design and long follow-up RCTs to confirm the results of this study.

## Author contributions

**Conceptualization:** Fei Wang, Ying Zhang.

**Funding acquisition:** Fei Wang.

**Investigation:** Liping Tang, Lingxia Xu, Jiangfeng Wu.

**Methodology:** Yanping Wang, Mingru Huang.

**Writing – original draft:** Yanping Wang, Mingru Huang.

**Writing – review & editing:** Fei Wang, Ying Zhang.
